# The mediating effects of home environment on the relationship between maternal BMI and childhood obesity: Findings from the ECHO-wide cohort

**DOI:** 10.1007/s00431-026-07368-6

**Published:** 2026-09-11

**Authors:** Divya Nair Haridas, Angham Ibrahim Tartour, Onno C. P. van Schayck, Marla T. H. Hahnraths, N. Sreekumaran Nair, Giridhara R. Babu

**Affiliations:** 1 Department of Family Medicine, Care and Public Health Research Institute (CAPHRI), Maastricht University, P.O. Box 616, 6200 MD Maastricht, The Netherlands; 2 Department of Population Medicine, College of Medicine, QU Health, Qatar University, P. O. Box: 2713, Doha, Qatar; 3 Department of Biostatistics, Jawaharlal Institute of Postgraduate Medical Education & Research, Puducherry, 605006 India

**Keywords:** Home environment, Adiposity, Children, Body mass index

## Abstract

Maternal pre-pregnancy obesity could affect long-term excessive child weight gain. Positive home environments, encompassing supportive social settings within a household, may mitigate such effects by promoting engagement in healthier behaviours. However, the mediating role of home environments is not established. The study examined whether nurturing and stimulating home environments mediate the association between maternal pre-pregnancy body mass index (BMI) and BMI z-score in early childhood (3–6 years of age). A retrospective cohort study of mother–child pairs using data from the Environmental Influences on Child Health Outcomes (ECHO) program in the USA was conducted, with data collected between September 2016 and March 2023 in children born between 2002 and 2011. The home environment was assessed using the Infant–Toddler Home Observation for Measurement of Environment (IT-HOME) questionnaire, with the Parental Responsivity, Acceptance, Learning Materials, Organisation, Variety and Parental Involvement subscales. Structural equation modelling (SEM) was used to test the potential mediating effects of IT-HOME subscales. The final analytic sample comprised 1341 mother–child pairs. Children whose mothers were overweight and obese during pre-pregnancy had significantly higher BMI z-scores in early childhood as compared to those of mothers who had normal weight, across models examining the six IT-HOME subscales as well as the total IT-HOME z-score. The home environment did not mediate the association between maternal pre-pregnancy BMI and early childhood BMI z-score.

*Conclusion*: This study had insufficient evidence to show that the home environment acted as a mediator in reducing the childhood BMI z-score associated with maternal pre-pregnancy BMI. Other dimensions, such as quality diet and physical activity stimulation, should be further explored as potential mediators in future studies.

**Table:** 

**What is Known:** • *To the best of our knowledge, this is the first study to look at the mediation effect of home environment in the relationship between childhood obesity and maternal pre-pregnancy BMI in 3–6-year-olds in the USA population.*
**What is New:** • *There was no evidence that a supportive and enriched home environment during infancy and toddlerhood acted as a mediator in attenuating the otherwise positive relationship between childhood obesity and maternal pre-pregnancy BMI in the USA population.* • *Future research should explore the mediating effects of dietary quality, physical activity dimensions, and the dynamics of age-specific effects of the home environment on weight trajectories across infancy, early childhood, middle childhood, and adolescence to have targeted interventions that are protective against childhood obesity.*

**Supplementary Information:**

The online version contains supplementary material available at https://doi.org/10.1007/s00431-026-07368-6.

## Introduction

Childhood overweight and obesity are major global health issues, impacting health, development, and life expectancy [1–3], with more than 390 million overweight children and adolescents aged 5–19 years worldwide [3]. In the United States of America (USA), 15.1 million children aged 5–14 years were overweight or obese in 2021 [4]. Childhood overweight often persists into adulthood, increasing risks of diabetes, cardiovascular diseases, impaired breathing, and cancer [5]. Obesity is also linked to depression, reduced quality of life, poorer health, victimisation, and discrimination, which can lead to psychological distress and can harm social, academic, and healthy behaviours [5–10]. Early-life growth patterns are predictive of later obesity [11]; infants with rapid weight gain in the first 2 years of life have a higher risk of later obesity [12]. Additionally, most of the pre-puberty excess weight is accumulated before the age of five [12], necessitating an understanding and addressing these factors in this critical developmental window.

Multiple factors may shape early uncontrolled weight gain trajectories, including high maternal pre-pregnancy body mass index (BMI), maternal prenatal tobacco exposure, greater infant birthweight, low socio-economic status, genetic predisposition, low physical activity levels, and no breastfeeding [13–28]. However, limited research has explored factors that may affect the association between maternal pre-pregnancy BMI and childhood adiposity, particularly the role of the home environment.

Prior research, albeit limited suggests that the home environment may influence early childhood obesity through behavioural and biological pathways after 6 years [29]. A plausible mechanism is that poorer home environments may be linked to lower adiponectin levels [30–33], a hormone that regulates adipose tissue expansion and may contribute to obesity [34]. Also, positive parent–child relationships may promote self-regulatory skills, including food intake regulation, and can protect against obesity [35, 36]. Children engaging in enriching activities and parent–child interaction demonstrate lower BMI percentiles [37]. These mechanisms support exploration of the home environment’s mediating role in the risk of early childhood obesity.

The home environment encompasses the child’s experience, encouragement, and support at home and impacts health outcomes [38, 39]. Although maternal pre-pregnancy BMI is linked with childhood obesity, its influence when considering the home environment as a mediator remains unclear. Using the prospective data from the Environmental Influences on Child Health Outcomes (ECHO) program [40], it was hypothesised that a nurturing and stimulating infant–toddler home environment mitigates the association of maternal pre-pregnancy BMI with BMI z-score in children aged 3–6 years by promoting healthier behavioural regulation and protecting against early biological risk for excess weight gain. Hence, the present study sought to answer the following research questions:

1. Is maternal pre-pregnancy BMI associated with BMI z-score at 3–6 years of life in the USA population?
2. Does infant–toddler home environment mediate the association between maternal pre-pregnancy BMI and BMI z-score at 3–6 years of life in the USA population?

## Materials and methods

A retrospective cohort study was conducted using data from the ECHO program, 2nd release [41]. The ECHO program is a nationwide research initiative spanning 44 USA states, designed to investigate the impact of diverse early-life environmental exposures on child health and developmental outcomes. ECHO comprises 69 cohorts and more than 50,000 infants, children, teens, and their families [40, 42], with data collected between September 2016 and March 2023. Participants in the current study were singleton infants/toddlers born at term (≥ 37 completed weeks of gestation) between 2002 and 2011, with anthropometric data available during early childhood (3–6 years of age) from two de-identified cohorts. Participants were excluded if they were born from a multiple-birth pregnancy, as multiple fetuses are an established risk factor for preterm birth and low birth weight [43], or had no available data on the Infant–Toddler Home Observation for Measurement of Environment (IT-HOME) or early childhood BMI z-score. The infant–toddler age window at outcome measurement was 3–6 years, and that of the mediator (IT-HOME) was 0–3 years. The local institutional review board approved the data-collection protocol for each cohort, and participants provided written informed consent. Confidentiality of the data was maintained by the ECHO data analysis centre. This study used deidentified data obtained from the NICHD Data and Specimen Hub (DASH).

### Exposure

The maternal pre-pregnancy BMI was categorised as underweight (< 18.5 kg/m^2^), normal (18.5–24.9 kg/m^2^), overweight (25.0–29.9 kg/m^2^), and obese (≥ 30.0 kg/m^2^) [44]. Maternal pre-pregnancy BMI (kg/m^2^) was either self-reported or calculated using the pre-pregnancy weight and maternal height, obtained either by direct measurement or from medical records. Data on pre-pregnancy BMI were collected at a single point after the last menstrual period and prenatally.

### Mediator

Trained personnel completed the IT-HOME questionnaire by observing parent–child interactions in the home environment and conducting semi-structured interviews with caregivers of infants and toddlers [39] aged 0–3 years. The IT-HOME questionnaire includes 45 items across six subscales, with two possible responses: yes (score = 1) or no (score = 0) [45]. A higher total IT-HOME score indicates a healthier home environment. The six subscales of the IT-HOME are as follows: (1) emotional and verbal responsiveness of the primary caregiver (hereafter called “parental responsivity”), refers to communication and emotional interactions between the parent and child (11 items); (2) avoidance of restriction and punishment (hereafter called “acceptance”), addresses how the primary caregiver disciplines the child and includes eight items; (3) organisation of the physical and temporal environment (hereafter called “organisation”), refers to the way of organising the child’s time outside the house and what the child’s personal space looks like (6 items); (4) providing appropriate play materials (hereafter called “learning materials”), consists of nine items that examine the presence of various types of toys available to children that are suitable for their age; (5) parental involvement with the child (hereafter called “parental involvement”), examines the physical interaction of the caregiver with the child (6 items); and (6) opportunities for variety in daily stimulation (hereafter called “variety”), refers to the designing of child’s daily routine in a way to incorporate social meetings with people other than the mother (6 items) [38, 46]. The questionnaire was validated in the USA population, with an internal consistency ranging from 0.44 to 0.89 suggesting moderate to strong internal consistency with moderate test–retest reliability and 90% between observer agreement [39, 47]. In this study, all six subscales, as well as the total IT-HOME z-score were considered, as mediators. Total scores for each of the six subscales were obtained. Standardisation was performed by rescaling the total subscale scores to follow a standard normal distribution (mean = 0 and standard deviation = 1). A total IT-HOME z-score was calculated by adding the six standardised subscale scores.

### Outcome

Age and sex specific BMI *z*-scores were calculated through CDC or WHO program depending on whether the children were between 24 and 239 months of age or under 24 months of age [48, 49]. Weight and height were obtained using one of the following methods: parent-report, medical record, or physical assessments. For this study, BMI z-scores of children in the age group of 3–6 years were used.

### Covariates

**Fig. 1:**
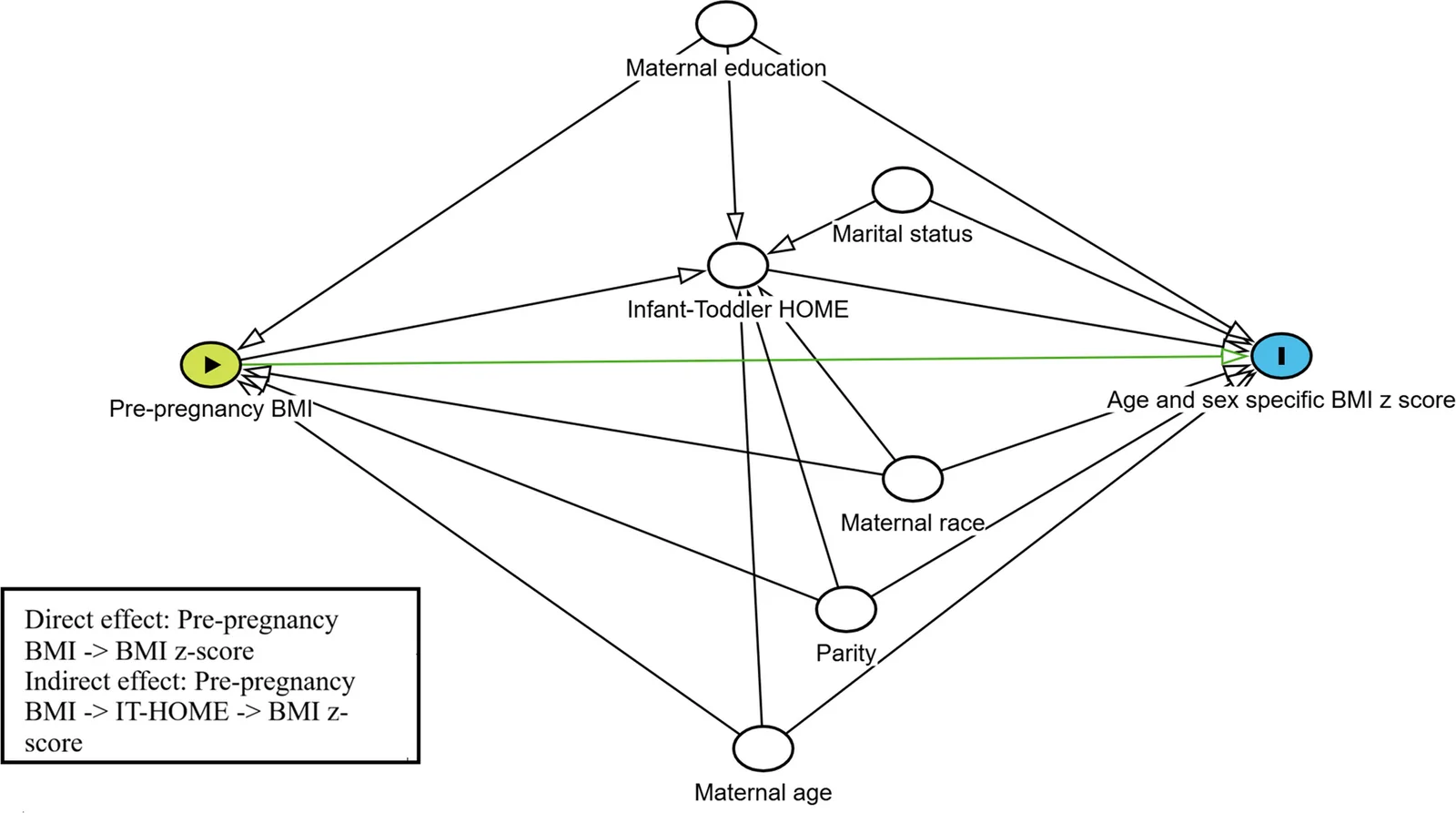
Directed acyclic graph depicting the association between pre-pregnancy BMI and early childhood body mass index z-score

A comprehensive literature search was conducted, and directed acyclic graphs were generated to identify the minimal adjustment set of confounders for examining the relationships between maternal pre-pregnancy BMI and early childhood BMI z-score (Fig. 1) [50]. Eight confounders were identified, including maternal marital status, maternal education, alcohol consumption during pregnancy, smoking during pregnancy, socio-economic status, maternal age at birth of the child (in years), parity, and maternal race [51–61]. Similarly, for the mediator-outcome association and exposure-mediator association, the identified confounders were maternal education, maternal age, parity, maternal race, marital status [34, 62–65] and maternal education, maternal age, parity and maternal race [66–69], respectively. Socio-economic status was calculated as the percentage of total combined income of the household during the last calendar year compared to the federal poverty level, according to the total number of household members and the number of children under 18 years old. This was further categorised as <  = 130%, > 130% to 350%, and > 350% of federal poverty levels. Maternal education was categorised as “less than high school”, “high school degree, general educational development or equivalent”, “some college, no degree; associate’s degree; trade school”, “bachelor’s degree”, and “master’s; professional or doctorate degree”, marital status as “married or living with a partner”, “widowed; separated; divorced”, and “single, never married; Partnered not living together”. Presence (yes) or absence (no) of smoked cigarettes and alcohol consumed during pregnancy was recorded. Maternal race was categorised as White, Black, Multiple Races, and other races (including Asian, Native Hawaiian or Pacific Islander, American Indian or Alaska Native, and other races). Parity was defined as the number of pregnancies carried to > 20 weeks of gestation, excluding the ECHO Index pregnancy. Smoking and alcohol use during pregnancy, and socio-economic status were excluded as follows, (1) these variables were in the causal pathway between maternal education and weight-for-height z score, (2) maternal education was associated with both pre-pregnancy BMI (exposure) and BMI z-score (outcome).

### Statistical analysis

Structural equation modelling (SEM) estimated direct and indirect effects of maternal pre-pregnancy BMI on early childhood BMI z-score. Separate single-mediator models were fitted for each IT-HOME subscale and the total IT-HOME z-score. Each model estimated (i) the direct effect, defined as the effect of maternal pre-pregnancy BMI on early childhood BMI z-score that is not transmitted through the mediator, (ii) the indirect effect, defined as the component of the effect transmitted through the IT-HOME mediator, and (iii) the total effect, defined as the sum of the direct and indirect effects. Parameter estimates were obtained using full-information maximum likelihood estimation (FIML) with robust (Huber–White) standard errors. Indirect and total effects with 95% confidence intervals (CIs) were derived using the delta method. Significance was assessed using 95% CIs and Sobel z-tests (*α* = 0.05).

Results are reported as unstandardised adjusted regression coefficients (β) with 95% CIs and *p*-values. SEM was performed using continuous maternal pre-pregnancy BMI as exposure. Unstandardised and standardised effect estimates with 95% CIs and *p*-values are presented in the Online Resource 1. Unstandardised regression effect estimates represent change in BMI z-score per one-unit increase in the predictor and standardised effect estimates represent change in standard deviation (SD) of BMI z-score per one-SD increase in the predictor. Exposure-to-mediator and mediator-to-outcome coefficients with 95% CI are reported in the Online Resource 2. Analyses utilised the earliest time point with complete data to reduce bias and each IT-HOME assessment preceded the corresponding BMI measurement. As the primary reduction in sample size is structural (subset data collection) rather than dropout-driven, Little’s MCAR test was not formally applied. All analyses were performed using Stata version 19.5. Model equations and related information are provided in Online Resource 3.

## Results

**Fig. 2:**
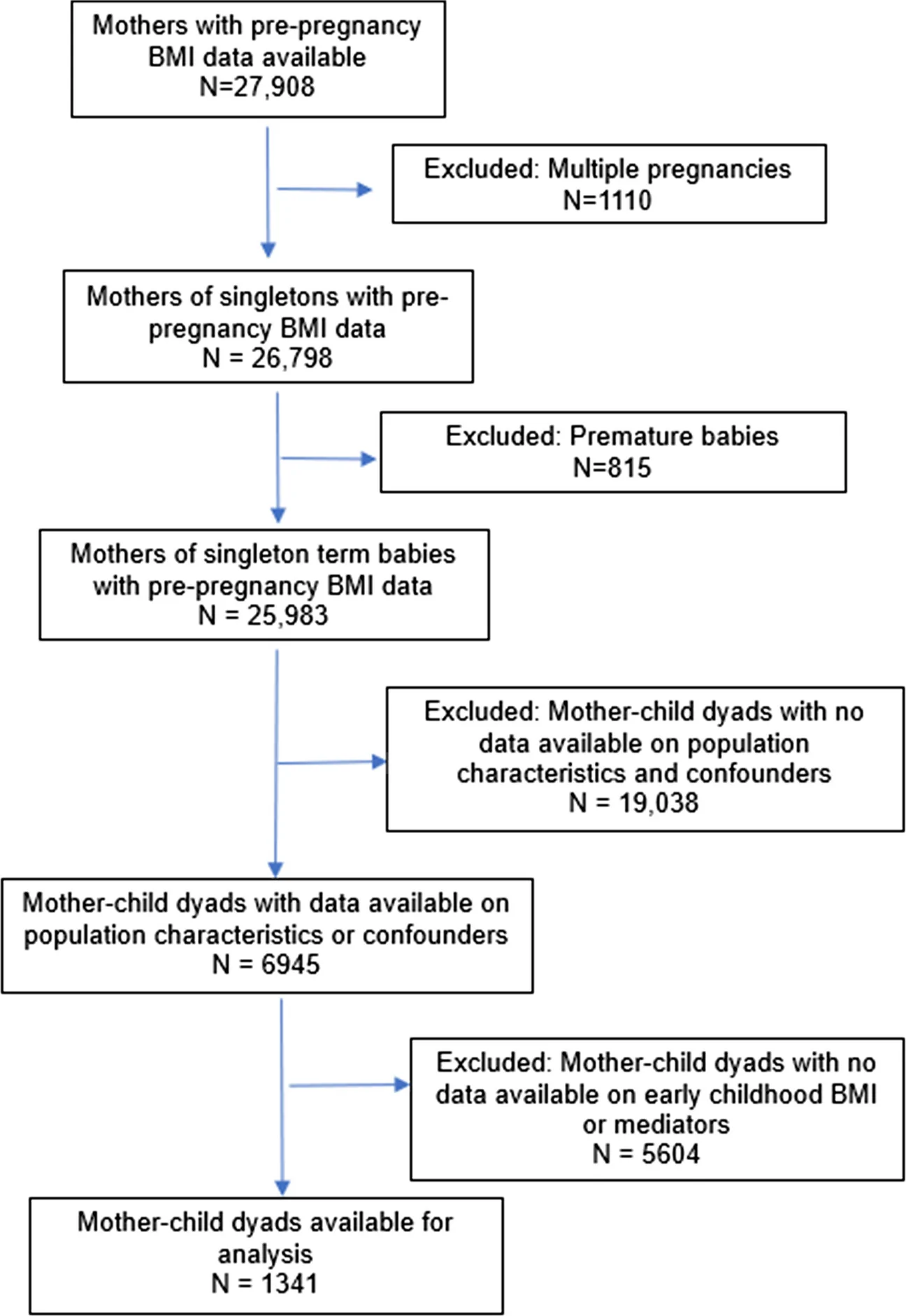
Flowchart of the study population based on maternal pre-pregnancy BMI exposure

Out of 27,908 mother–child dyads with maternal pre-pregnancy data, a total of 6945 had one or more baseline characteristics available (Fig. 2). These numbers varied further, given the availability of IT-HOME data (*n* = 1341), as IT-HOME data were collected for a subset of the study population with one or more baseline characteristics available (Fig. 2) comprising 19.3% of the dyads.

Table 1 compares demographic and exposure characteristics of dyads with and without IT-HOME data; although the two groups were broadly comparable in their pre-pregnancy BMI distribution, they differed in maternal age, race, marital status, and educational attainment, indicating that the data were unlikely to be missing completely at random. Missingness was therefore assumed to be at random conditional on the DAG-identified covariates (maternal education, maternal age at birth of the child, parity, maternal race and marital status), all of which were adjusted for in the analyses. The remaining characteristics presented in Table 1, including child sex and child race, are reported descriptively to characterise the analytic sample and were not part of the minimal adjustment set identified by the directed acyclic graph. Residual selection bias cannot be excluded. Most mothers included in the analysis (43.5%) held a high school degree, General Educational Development or equivalent, and 60.3% were married or living with a partner and had normal pre-pregnancy BMI (41.4%). The sample was predominantly composed of White and Black children, who were represented in approximately equal proportions (44.3%) and 52.2% were males. The median age of the child at measurement of IT-HOME was 0.8 years (Q1-Q3, 0.7–2.0). The mean birthweight was 3.3 kg (SD = 0.5). At follow-up (3–6 years of age), children’s mean age was 3.8 years (SD = 0.8) and mean BMI z-score was 0.5 (SD = 1.3).

**Table 1:** Population characteristics, anthropometrics, and exposure characteristics of dyads excluded from and included in the analysis based on the availability of IT-HOME data in the environmental influences on child health outcomes-wide cohort

Variables	Dyads excluded from the analysis (*N* = 5599) *n* (%)	Dyads included in the analysis (*N* = 1341) *n* (%)
**Maternal education level**	*N* = 3494	*N* = 566
Less than high school	263 (7.5)	48 (8.5)
High school degree, general educational development, or equivalent	483 (13.8)	246 (43.5)
Some college, no degree; associate’s degree; trade school	902 (25.8)	54 (9.5)
Bachelor’s degree	1,047 (30.0)	133 (23.5)
Master’s, professional, or doctorate degree	799 (22.9)	85 (15.0)
**Age of mother (years)**	*N* = 5485 29.9 (5.4)^*^	*N* = 1341 26.2 (5.9)^*^
**Maternal marital status**	*N* = 3693	*N* = 566
Married or living with a partner	3042 (82.4)	341 (60.3)
Widowed; separated; divorced	119 (3.2)	18 (3.2)
Single, never married; partnered, not living together	532 (14.4)	207 (36.6)
**Maternal race**	*N* = 5701	*N* = 1312
White	3770 (66.1)	661 (50.4)
Black	1083 (19)	566 (43.1)
Multiple race	646 (11.3)	57 (4.3)
Others	202 (3.5)	28 (2.1)
**Maternal pre-pregnancy BMI**	*N* = 5404	*N* = 1030
Underweight	149 (2.8)	47 (4.6)
Normal	2208 (40.9)	426 (41.4)
Overweight	1407 (26.0)	257 (25.0)
Obese	1640 (30.4)	300 (29.1)
**Child sex**	*N* = 5600	*N* = 1,341
Male	2821 (50.4)	700 (52.2)
Female	2779 (49.6)	641 (47.8)
**Child race**	*N* = 5215	*N* = 1338
White	3301 (63.3)	592 (44.3)
Black	953 (18.3)	593 (44.3)
Multiple race	436 (8.4)	16 (1.2)
Others	525 (10.1)	137 (10.2)
Parental responsivity subscale total score (range 0–11)	-	*N* = 1180 9.6 (1.8)
Acceptance subscale total score (range 0–8)	-	*N* = 1176 6.9 (1.2)
Organisation subscale total score (range 2–6)	-	*N* = 868 4.9 (1.1)
Learning materials subscale total score (range 0–9)	-	*N* = 1283 7.7 (1.5)
Parental involvement subscale total score (range 0–6)	-	*N* = 834 5.2 (1.1)
Variety subscale total score (range 1–5)	-	*N* = 870 4.2 (0.9)
Total IT-HOME z-score	-	*N* = 695 0.3 (3.1)
BMI z-score	*N* = 1701 0.4 (1.5)^*^	*N* = 1186 0.5 (1.3)^*^

^*^Mean (SD)

On average, families’ scores approached the higher end of the scales across all IT-HOME subscales, reflecting an enriching home environment (Table 1).

### Maternal pre-pregnancy BMI and early childhood BMI z-score (direct effect)

Table 2 summarises the direct, indirect, and total effects for each IT-HOME subscale and for the total IT-HOME z-score; each row corresponds to a separate single-mediator model. Children of mothers who were overweight and obese during the pre-pregnancy period had higher BMI z-scores as compared to children of mothers who had a normal weight, across models examining the six IT-HOME subscales as well as the total IT-HOME z-score. Being in the underweight category did not affect the early childhood BMI z-score (Table 2).

**Table 2:** Direct, indirect, and total effects of maternal pre-pregnancy BMI categories on BMI z-score, mediated through home environment subscales (infant–toddler home observation of measurement of environment subscales and total infant–toddler home observation of measurement of environment z-score)

	Direct effect (maternal pre-pregnancy body mass index categories on BMI z-score)			Indirect effect (via mediator)			Total effect (maternal pre-pregnancy body mass index categories on BMI z-score)		
IT-HOMEa mediator	Underweight	Overweight	Obese	Underweight	Overweight	Obese	Underweight	Overweight	Obese
Acceptance	β = − 0.3 (95% CI: − 0.8 to 0.1), *p* = 0.166	β = 0.4 (95% CI: 0.2 to 0.6), *p* < 0.001*	β = 0.6 (95% CI: 0.4 to 0.8), *p* < 0.001*	β = − 0.0 (95% CI: − 0.0 to 0.0), *p* = 0.416	β = 0.0 (95% CI: − 0.0 to 0.0), *p* = 0.965	β = 0.0 (95% CI: − 0.0 to 0.0), *p* = 0.753	β = − 0.3 (95% CI: − 0.8 to 0.1), *p* = 0.157	β = 0.4 (95% CI: 0.2 to 0.6), *p* < 0.001*	β = 0.6 (95% CI: 0.4 to 0.8), *p* < 0.001*
Learning materials	β = − 0.3 (95% CI − 0.8 to 0.1), *p* = 0.165	β = 0.4 (95% CI: 0.2 to 0.6), *p* < 0.001*	β = 0.6 (95% CI: 0.4 to 0.8), *p* < 0.001*	β = − 0.0 (95% CI: − 0.0 to 0.0), *p* = 0.971	β = 0.0 (95% CI: − 0.0 to 0.0), *p* = 0.971	β = 0.0 (95% CI: − 0.0 to 0.0), *p* = 0.971	β = − 0.3 (95% CI: − 0.8 to 0.1), *p* = 0.164	β = 0.4 (95% CI: 0.2 to 0.6), *p* < 0.001*	β = 0.6 (95% CI: 0.4 to 0.8), *p* < 0.001*
Parental responsivity	β = − 0.3 (95% CI: − 0.8 to 0.1), *p* = 0.160	β = 0.4 (95% CI: 0.2 to 0.6), *p* < 0.001*	β = 0.6 (95% CI: 0.4 to 0.8), *p* < 0.001*	β = 0.0 (95% CI: − 0.0 to 0.0), *p* = 0.971	β = − 0.0 (95% CI: − 0.0 to 0.0), *p* = 0.968	β = 0.0 (95% CI: − 0.0 to 0.0), *p* = 0.969	β = − 0.3 (95% CI: − 0.8 to 0.1), *p* = 0.160	β = 0.4 (95% CI: 0.2 to 0.6), *p* < 0.001*	β = 0.6 (95% CI: 0.4 to 0.8), *p* < 0.001*
Organisation	β = − 0.3 (95% CI: − 0.8 to 0.1), *p* = 0.166	β = 0.4 (95% CI: 0.2 to 0.6), *p* < 0.001*	β = 0.6 (95% CI: 0.4 to 0.8), *p* < 0.001*	β = 0.0 (95% CI: − 0.0 to 0.0), *p* = 0.985	β = 0.0 (95% CI: − 0.0 to 0.0), *p* = 0.985	β = − 0.0 (95% CI: − 0.0 to 0.0), *p* = 0.985	β = − 0.3 (95% CI: − 0.8 to 0.1), *p* = 0.166	β = 0.*4* (95% CI: 0.2 to 0.6), p < 0.001*	β = 0.6 (95% CI: 0.4 to 0.8), *p* < 0.001*
Variety	β = − 0.3 (95% CI: − 0.8 to 0.1), *p* = 0.157	β = 0.4 (95% CI: 0.2 to 0.6), *p* < 0.001*	β = 0.6 (95% CI: 0.4 to 0.8), *p* < 0.001*	β = − 0.0 (95%CI: − 0.0 to 0.0), *p* = 0.881	β = − 0.0 (95% CI: − 0.0 to 0.0), *p* = 0.824	β = 0.0 (95% CI: − 0.0 to 0.0), *p* = 0.849	β = − 0.3 (95% CI: − 0.8 to 0.1), *p* = 0.157	β = 0.4 (95% CI: 0.2 to 0.6), *p* < 0.001*	β = 0.6 (95% CI: 0.4 to 0.8), *p* < 0.001*
Parental involvement	β = − 0.3 (95% CI: − 0.8 to 0.1), *p* = 0.164	β = 0.4 (95% CI: 0.2 to 0.6), *p* < 0.001*	β = 0.6 (95% CI: 0.4 to 0.8), *p* < 0.001*	β = − 0.0 (95% CI: − 0.0 to 0.0), *p* = 0.648	β = 0.0 (95% CI: − 0.0 to 0.0), *p* = 0.660	β = 0.0 (95% CI: − 0.0 to 0.0), *p* = 0.535	β = − 0.3 (95% CI: − 0.8 to 0.1), *p* = 0.160	β = 0.4 (95% CI: 0.2 to 0.6), *p* < 0.001*	β = 0.6 (95% CI: 0.4 to 0.8), *p* < 0.001*
Total IT-HOME z-score	β = − 0.3 (95% CI: − 0.8 to 0.1), *p* = 0.179	β = 0.4 (95% CI: 0.2 to 0.6), *p* < 0.001*	β = 0.6 (95% CI: 0.4 to 0.8), *p* < 0.001*	β = − 0.0 (95% CI: − 0.0 to 0.0), *p* = 0.291	β = 0.0 (95% CI: − 0.0 to 0.0), *p* = 0.743	β = 0.0 (95% CI: − 0.0 to 0.0), *p* = 0.821	β = − 0.3 (95% CI: − 0.8 to 0.1), *p* = 0.158	β = 0.4 (95% CI: 0.2 to 0.6), *p* < 0.001*	β = 0.6 (95% CI: 0.4 to 0.8), *p* < 0.001*

*p < 0.001

Reference category: normal weight

^a^Infant-toddler home observation of measurement of environment

Models adjusted for maternal marital status, maternal education, maternal age at birth of the child, parity and maternal race; mediator: IT-HOME subscales

### Mediation effect of home environment on the association between maternal pre-pregnancy BMI and early childhood BMI z-score (indirect effect)

The indirect effects through the IT-HOME domains and total IT-HOME z-score were not statistically significant (Table 2). Table 3 provides the percentage of the association explained by the home environment subscales. These findings suggest that the home environment does not have a mediating effect on the association between maternal pre-pregnancy BMI and early childhood BMI z-score. In all models, the direct effect of maternal pre-pregnancy BMI on early childhood BMI z-score remained almost identical to the total effect, indicating a negligible mediating effect (Table 2). Standardised effect estimates obtained from the models with continuous exposure, also suggested no significant mediation effects (Online Resource 1).

**Table 3:** Contribution of home environment subscales (infant–toddler home observation of measurement of environment subscales and total infant–toddler home observation of measurement of environment z-score) to the association between maternal pre-pregnancy BMI categories and early childhood BMI z-score

IT-HOME^a^ mediator	Percentage mediated (%)		
	**Underweight**	**Overweight**	**Obese**
**Acceptance**	2.2	0.0	0.2
**Learning materials**	0.1	0.0	0.0
**Parental responsivity**	0.0	0.0	0.0
**Organisation**	0.0	0.0	0.0
**Variety**	0.1	0.1	0.0
**Parental involvement**	1.1	0.4	0.8
**Total IT-HOME z-score**	4.7	0.5	0.3

Reference category: normal weight

^a^Infant-toddler home observation of measurement of environment

Models adjusted for maternal marital status, maternal education, maternal age at birth of the child, parity and maternal race; mediator: IT-HOME subscales

Percentages mediated are ratios of the indirect to the total effect and are unstable when the total effect is small or not statistically significant; they should therefore be interpreted alongside the effect estimates presented in Table 2

## Discussion

This ECHO multi-cohort study found that children of mothers belonging to the overweight and obese categories of pre-pregnancy BMI had higher early childhood BMI z-score as compared to children of mothers belonging to the normal weight category, even after accounting for socio-demographic factors. There was insufficient evidence to suggest that the quality of the child’s early home environment mediated this relationship, whether maternal pre-pregnancy BMI was modelled categorically (Table 2) or as a continuous variable (Online Resource 1). No mediation effect was found among underweight, overweight, or obese mothers.

The findings align with prior research linking maternal pre-pregnancy BMI to higher child BMI and weight [60, 70–72]. Similarly, maternal pre-pregnancy overweight is a recognised risk factor for childhood obesity [73].

Additionally, high parental warmth (acceptance), learning opportunities (learning materials) and family integration (parental involvement) scores were associated with lower BMI. No association was found between BMI and parental responsivity [37]. Another study found high organisation of environment (organisation), learning material stimulation (learning materials), and acceptance child scores to lower BMI [74]. Tindula et al. found broader associations between overall HOME scores and BMI [34]. The present study did not identify the home environment as a mediator that reduces childhood BMI z-score. This is possibly due to the small analytic sample available for the analyses that might have limited the power to detect the mediation effect in the expected direction.

Supportive caregiving environments may encourage physical activity and the consumption of nutritious foods and help prevent binge-eating behaviours. For example, parental secure attachment was found to help children manage stress [75], possibly preventing eating as a stress response [76, 77]. Parents can also play a major role in preventing childhood obesity by encouraging and providing their children with opportunities for physical activity [78]. The home environment may also be reflected by parental feeding behaviours, which could affect child obesity and health outcomes. Parental use of encouragement and pressure to eat was found to decrease the odds of childhood overweight, whereas parental food restriction and permissive feeding practices increased the odds of overweight [79]. Furthermore, two studies found marked associations between greater parental pressure to eat and adverse eating outcomes, with one study reporting reduced daily fruit consumption [80] and another indicating increased levels of eating fussiness [81]. A non-responsive home feeding environment likely contributed to heightened levels of picky eating behaviour among preschool-aged children [82].

### Strengths and limitations

Strengths of this study include its diverse, multi-cohort sample and the prospective assessment of early risk factors and home environment in relation to BMI z-scores. Limitations include a small sample size and missing data, which may have limited the power to conduct a mediation analysis. Additionally, missing data may have introduced selection bias and affected the generalisability of the findings. Furthermore, the study could not incorporate factors (such as parental genetics, shared lifestyle factors, and diet), which may have introduced residual confounding. The home environment may change over time; however, the present study could not capture these changes, potentially influencing the strength and/or direction of the observed relationships. Maternal pre-pregnancy BMI and early childhood weight and height data were partly self-reported, which may have led to underestimation. Additionally, the exact timing of maternal pre-pregnancy weight measurement was unknown, likely introducing variation in BMI measurements, thereby influencing the effect estimates. Specifically, the ECHO-DASH database provided no information to distinguish measurement modes (parent-reported, measured, or medical record) for child weight, child height, or maternal pre-pregnancy BMI across cohorts. Variability in measurement protocols across contributing sites may have introduced non-differential misclassification bias. Additionally, a mixed-effects mediation model with random intercepts for cohort site did not converge, likely due to the small analytic sample.

## Conclusion

While biological factors, such as maternal pre-pregnancy BMI, were associated with early childhood BMI z-score, insufficient evidence showed that better supportive and enriching home environments during infancy and toddlerhood could lower the childhood BMI z-score associated with high maternal pre-pregnancy BMI. Future studies should explore the following: (1) mediating effect of home environment using a cohort study design with larger sample size; (2) the mediating effects of other dimensions, such as dietary quality, stimulation of physical activity, screen time, sleep patterns, and feeding practices, that are considered integral elements of the home environment in prior research [79, 82–84], with adequate time gap between the mediator and outcome measurements. Further research should also explore the age-specific effects of the home environment on weight trajectories across infancy, early childhood, middle childhood, and adolescence. Understanding these dynamics can inform targeted interventions to support caregivers in creating protective environments against childhood obesity.

## Supplementary Information

Below is the link to the electronic supplementary material.

ESM 1 (PDF 173 KB)

ESM 2 (PDF 109 KB) 

ESM 3 (PDF 145 KB)

## Data Availability

The data that support the findings of this study are available from the NICHD Data and Specimen Hub (DASH), but restrictions apply to their availability; these data were used under a data use agreement. The data are, however, available upon request and with the permission of the National Institutes of Health.

## Abbreviations

BMI: Body mass index
ECHO: Environmental influences on Child Health Outcomes
IT-HOME: Infant-Toddler Home Observation for Measurement of Environment
SEM: Structural equation modelling
FIML: Full-information maximum likelihood estimation
