# Supplementary material for: The mediating effects of home environment on the relationship between maternal BMI and childhood obesity: Findings from the ECHO-wide cohort
Source: Eur J Pediatr. 2026 Sep 11;185(10):740. doi: 10.1007/s00431-026-07368-6 (PMC13562283; doi:10.1007/s00431-026-07368-6)
Supplement: Supplementary file 1 [file 431_2026_7368_MOESM1_ESM.pdf]

Article title: The Mediating Effects of Home Environment on the Relationship between Maternal BMI and Childhood Obesity: Findings from the ECHO-wide Cohort

Journal name: European Journal of Pediatrics

Author names: Divya Nair Haridas, Angham Ibrahim Tartour, Onno C.P. van Schayck, Marla T.H. Hahnraths, N. Sreekumaran Nair, Giridhara R. Babu

Affiliations

Department of Family Medicine, University of Maastricht, Maastricht, Netherlands

Divya Nair Haridas, Onno C.P. van Schayck & Marla T.H. Hahnraths

Department of Population Medicine, College of Medicine, QU Health, Qatar University, Doha, Qatar

Angham Ibrahim Tartour & Giridhara R. Babu

Department of Biostatistics, Jawaharlal Institute of Postgraduate Medical Education & Research, Puducherry, India

N. Sreekumaran Nair

Corresponding author

Correspondence to Divya Nair Haridas

Direct, indirect, and total effects (standardised and unstandardised) of maternal pre-pregnancy BMI (continuous) on BMI z-score, mediated through home environment domains (infant-toddler home observation of measurement of environment subscales and total infant-toddler home observation of measurement of environment z-score).

	**Direct effect (maternal pre-pregnancy body mass index on BMI z-score)**		**Indirect effect (via mediators)**		**Total effect (maternal pre-pregnancy body mass index on BMI z-score)**	
**IT-HOME[a] Mediator**	**Unstandardised β**	**Standardised β**	**Unstandardised β**	**Standardised β**	**Unstandardised β**	**Standardised β**
**Acceptance**	β = 0.03 (95% CI: 0.02 to 0.04), p < 0.001*	β = 0.22 (95% CI: 0.14 to 0.30), p < 0.001*	β = 0.00 (95% CI: -0.00 to 0.00), p = 0.514	β = 0.00 (95% CI: -0.00 to 0.01), p = 0.514	β = 0.03 (95% CI: 0.02 to 0.04), p < 0.001*	β = 0.22 (95% CI: 0.14 to 0.30), p < 0.001*
**Learning Materials**	β = 0.03 (95% CI: 0.02 to 0.04), p < 0.001*	β = 0.22 (95% CI: 0.13 to 0.30), p < 0.001*	β = -0.00 (95% CI: -0.00 to 0.00), p = 0.989	β = -0.00(95% CI: -0.01 to 0.01), p = 0.989	β = 0.03(95% CI: 0.02 to 0.04), p < 0.001*	β = 0.22 (95% CI: 0.13 to 0.30), p < 0.001*
**Parental Responsivity**	β = 0.03 (95% CI: 0.02 to 0.04), p < 0.001*	β = 0.22 (95% CI: 0.13 to 0.30), p < 0.001*	β = -0.00(95% CI: -0.00 to 0.00), p = 0.793	β = -0.00 (95% CI: -0.00 to 0.00), p = 0.793	β = 0.03 (95% CI: 0.02 to 0.04), p < 0.001*	β = 0.22 (95% CI: 0.13 to 0.30), p < 0.001*
**Organisation**	β = 0.03 (95% CI: 0.02 to 0.04), p < 0.001*	β = 0.22 (95% CI: 0.14 to 0.30), p < 0.001*	β = 0.00 (95% CI: -0.00 to 0.00), p = 0.991	β = 0.00 (95% CI: -0.00 to 0.00), p = 0.991	β = 0.03 (95% CI: 0.02 to 0.04), p < 0.001*	β = 0.22 (95% CI: 0.14 to 0.30), p < 0.001*
**Variety**	β = 0.03 (95% CI: 0.02 to 0.04), p < 0.001*	β = 0.22 (95% CI: 0.13 to 0.30), p < 0.001*	β = 0.00 (95% CI: -0.00 to 0.00), p = 0.935	β = 0.00 (95% CI: -0.00 to 0.00), p = 0.935	β = 0.03 (95% CI: 0.02 to 0.04), p < 0.001*	β = 0.22 (95% CI: 0.13 to 0.30), p < 0.001*
**Parental involvement**	β = 0.03 (95% CI: 0.02 to 0.04), p < 0.001*	β = 0.21 (95% CI: 0.13 to 0.30), p < 0.001*	β = 0.00 (95% CI: -0.00 to 0.00), p = 0.593	β = 0.00 (95% CI: -0.01 to 0.01), p = 0.593	β = 0.03 (95% CI: 0.02 to 0.04), p < 0.001*	β = 0.22 (95% CI: 0.13 to 0.30), p < 0.001*
**Total IT-HOME[a] z-score**	β = 0.03 (95% CI: 0.02 to 0.04), p < 0.001*	β = 0.21 (95% CI: 0.13 to 0.29), p < 0.001*	β = 0.00 (95% CI: -0.00 to 0.00), p = 0.340	β = 0.00 (95% CI: -0.00 to 0.01), p = 0.340	β = 0.03 (95% CI: 0.02 to 0.04), p < 0.001*	β = 0.22 (95% CI: 0.13 to 0.30), p < 0.001*

*p < 0.001

[a]Infant-toddler home observation of measurement of environment

Models adjusted for maternal marital status, maternal education, maternal age at birth of the child, parity and maternal race; mediator: IT-HOME domains

Results are reported to two decimal places
