# Supplementary material for: The mediating effects of home environment on the relationship between maternal BMI and childhood obesity: Findings from the ECHO-wide cohort
Source: Eur J Pediatr. 2026 Sep 11;185(10):740. doi: 10.1007/s00431-026-07368-6 (PMC13562283; doi:10.1007/s00431-026-07368-6)
Supplement: Supplementary file 3 [file 431_2026_7368_MOESM3_ESM.pdf]

Article title: The Mediating Effects of Home Environment on the Relationship between Maternal BMI and Childhood Obesity: Findings from the ECHO-wide Cohort

Journal name: European Journal of Pediatrics

Author names: Divya Nair Haridas, Angham Ibrahim Tartour, Onno C.P. van Schayck, Marla T.H. Hahnraths, N. Sreekumaran Nair, Giridhara R. Babu

Affiliations

Department of Family Medicine, University of Maastricht, Maastricht, Netherlands

Divya Nair Haridas, Onno C.P. van Schayck & Marla T.H. Hahnraths

Department of Population Medicine, College of Medicine, QU Health, Qatar University, Doha, Qatar

Angham Ibrahim Tartour & Giridhara R. Babu

Department of Biostatistics, Jawaharlal Institute of Postgraduate Medical Education & Research, Puducherry, India

N. Sreekumaran Nair

Corresponding author

Correspondence to Divya Nair Haridas

SEM model equations and related information

Exposure: Pre-pregnancy BMI (X)

Outcome: Early childhood BMI z-score (Y)

Mediator: IT-HOME subscale scores (Parental Responsivity, Acceptance, Organisation, Learning Materials, Parental Involvement, Variety) and total IT-HOME z-score (considered one domain score at a time) (M)

Model equations

$$M = \beta_{0M} + \text{aX} + \beta_1 Maternal\ age\ at\ birth\ of\ the\ child + \beta_2 Parity + \beta_3 Maternal\ race + \beta_4 Maternal\ marital\ status + \beta_5 Maternal\ education\ level + e_M$$

$$Y = \beta_{0Y} + \text{cX} + bM + \beta_1 Maternal\ age\ at\ birth\ of\ the\ child + \beta_2 Parity + \beta_3 Maternal\ race + \beta_4 Maternal\ marital\ status + \beta_5 Maternal\ education\ level + e_Y$$

where,

a = effect of X on M

b = effect of M on Y conditional on exposure and covariates

c = effect of X on Y conditional on mediator and covariates

β_{0M} and β_{0Y} are the intercepts, $\beta_1, \beta_2, \beta_3, \beta_4, \beta_5$ are the slopes and e_M, e_Y are the residual errors

Distribution: Gaussian

Link: Gaussian

Calculation of indirect and total effects

Direct effect: c

Indirect effect: a*b

Total effect: c + a*b
